# An improved transmissibility model to detect transgenerational transmitted environmental effects

**DOI:** 10.1186/s12711-023-00833-y

**Published:** 2023-09-21

**Authors:** Ingrid David, Anne Ricard

**Affiliations:** 1 GenPhySE, Université de Toulouse, INRAE, ENVT, 31326 Castanet Tolosan, France; 2 INRAE, AgroParisTech, GABI, Université Paris Saclay, 78350 Jouy-en-Josas, France; 3 Département Recherche et Innovation, Institut Français du Cheval et de l’équitation, 61310 Exmes, France

## Abstract

**Background:**

Evolutionary studies have reported that non-genetic information can be inherited across generations (epigenetic marks, microbiota, cultural inheritance). Non-genetic information is considered to be a key element to explain the adaptation of wild species to environmental constraints because it lies at the root of the transgenerational transmission of environmental effects. The “transmissibility model” was proposed several years ago to better predict the transmissible potential of each animal by taking these diverse sources of inheritance into account in a global transmissible potential. We propose to improve this model to account for the influence of the environment on the global transmissible potential as well. This extension of the transmissibility model is the “transmissibility model with environment” that considers a covariance between transmissibility samplings of animals sharing the same environment. The null hypothesis of “no transmitted environmental effect” can be tested by comparing the two models using a likelihood ratio test (LRT).

**Results:**

We performed simulations that mimicked an experimental design consisting of two lines of animals with one exposed to a particular environment at a given generation. This enabled us to evaluate the performances of the transmissibility model with environment so as to detect and quantify transgenerational transmitted environmental effects. The power and the realized type I error of the LRT were compared to those of a T-test comparing the phenotype of the two lines, three generations after the environmental exposure for different sets of parameters. The power of the LRT ranged from 45 to 94%, whereas that of the T-test was always lower than 26%. In addition, the realized type I error of the T-test was 15% and that of the LRT was 5%, as expected. Variances, the covariance between transmissibility samplings, and path coefficients of transmission estimated with the transmissibility model with environment were close to their true values for all sets of parameters.

**Conclusions:**

The transmissibility model with environment is effective in modeling vertical transmission of environmental effects.

**Supplementary Information:**

The online version contains supplementary material available at 10.1186/s12711-023-00833-y.

## Background

Over the past decades, a growing body of research has shown that sources of information other than genetics are inherited across generations [1–4]. These non-genetic inherited sources of information are transmitted across generations via a physical transmission support, as is the case for epigenetic marks [5], microbiota [6] and thought learning mechanisms (behavioral/cultural inheritance [7, 8]). As opposed to DNA, non-genetic information sources are susceptible to be modified by the environment. For example, various studies have shown altered behavior and changes in epigenetic marks in mice whose ancestors experienced a stressful environment early in life [9]. This environmental sensitivity makes non-genetic information a key element in the explanation of the adaptation of wild species to environmental constraints [10–12]. Due to global warming, societal demands and the agroecological transition, farm animals will face new farming conditions, which will be mainly characterized by more variability in environmental conditions and feed resources, to which they will have to adapt. In response to this new challenge, genetic studies that propose new criteria for robustness, resilience and efficiency have increased [13–15]. Genetic improvement of these traits would make it possible to meet the new environmental constraints. Nonetheless, although genomic selection [16] can improve annual genetic gain [17], the genetic improvement of a population is a slow process that will not allow us to respond quickly enough to the new constraints. Considering non-genetic inherited effects for the selection of future reproducers by mimicking natural adaptive processes may overcome this difficulty [18]. Indeed, from the point of view of the inclusive evolutionary synthesis, non-genetic vertically transmitted effects make it possible to transfer recently acquired information about the current environment to offspring, facilitating adaptation [19]. The transmissibility model has been developed to account for genetic and non-genetic inheritance in the estimation of the global transmissible potential of individuals [20, 21]. Using phenotype and pedigree information, this model makes it possible to determine whether or not there is a significant proportion of inheritance of non-genetic origin in the vertical transmission of traits [22], but does not consider the impact of the environment on non-genetic inherited factors. In order to be able to evaluate the adaptation to which environmental conditions are transmitted across generations, our aim is to further develop the transmissibility model to include the quantification of transgenerational transmitted environmental effects. After a brief overview of the transmissibility model, its expansion to include transmissible environmental effects is presented, as well as its validation on simulated data.

## Methods

### Improved transmissibility model including environmental effects

As a reminder, the transmissibility model proposed by David and Ricard [21] is as follows:

1 $$y_{i} = {\mathbf{x}}_{{\mathbf{i}}} {{\varvec{\upbeta}}} + t_{i} + e_{i} ,$$

where ${y}_{i}$ is the phenotype of individual $i$, ${\varvec{\upbeta}}$ is the vector of fixed effects with known incidence vector ${\mathbf{x}}_{\mathbf{i}}$, ${t}_{i}$ is the “global transmissible potential” of animal $i$ that combines its different transmissible values (genetic, epigenetic, microbiote and culture [23–25]) with $\mathbf{t}\sim MVN\left(0,\mathbf{M}{\sigma }_{t}^{2}\right)$, where $\mathbf{M}$ is the matrix of transmission between individuals (transmission relationship matrix), ${\sigma }_{t}^{2}$ is the variance of the global transmissible potential, ${e}_{i}$ is the residual where $\mathbf{e}\sim MVN\left(0,\mathbf{I}{\sigma }_{e}^{2}\right),$ and $\mathbf{I}$ is the identity matrix. The model of transmission of the global transmissible potential is: ${t}_{i}={{\omega }_{s}t}_{si}+{{\omega }_{d}t}_{di}+{\varepsilon }_{i},$ where ${t}_{si}$ and ${t}_{di}$ are the global transmissible potential of the sire and dam of animal $i$, and ${\omega }_{s}$ and ${\omega }_{d}$ are the unknown path coefficients of transmission from the sire and the dam, respectively, that conform to the following constraints: $0\le {\omega }_{s}\le 1, 0\le {\omega }_{d}\le 1,$ $0\le {\omega }_{s}+{\omega }_{d}\le 1$. ${\varepsilon }_{i}$ refers to the transmissibility sampling with ${\varvec{\upvarepsilon}}\sim N\left({\varvec{0}},\mathbf{D}{\sigma }_{t}^{2}\right),$ where $\mathbf{D}$ is a diagonal matrix with variances of $\varepsilon$ relative to ${\sigma }_{t}^{2}$ as components ${(\delta }_{i})$, i.e., considering no inbreeding ${\delta }_{i}=\left(1-{\omega }_{s}^{2}-{\omega }_{d}^{2}\right)$ if both parents of animal $i$ are known, ${\delta }_{i}=\left(1-{\omega }_{d}^{2}\right)$ for animals of an unknown sire, ${\delta }_{i}=\left(1-{\omega }_{s}^{2}\right)$ for animals of an unknown dam, and 1 for animals for which both parents are unknown. Given this model of transmission, the inverse of the transmission relationship matrix can be easily obtained by the following decomposition: ${\mathbf{M}}^{{\varvec{-1}}}={\mathbf{L}}^{\mathbf{^{\prime}}}{\mathbf{D}}^{{\varvec{-1}}}\mathbf{L}$, where $\mathbf{L}$ is a lower triangular matrix with 1s on the diagonal and the negatives of the sire and dam’s coefficients of transmission as off-diagonal entries [26, 27]. Co-variance parameters of the transmissibility model (${\omega }_{s},{\omega }_{d}, {\sigma }_{t}^{2}, {\sigma }_{e}^{2})$ can be obtained using the ASReml software [28] and the program developed by David [20].

To improve the transmissibility model in order to account for the influence of the environment on the global transmissible potential (i.e., transgenerational transmitted environmental effect), we propose to modify the model of transmission of the global transmissible potential by including the impact of the environment in the transmissibility sampling. Thus, the transmissibility sampling is now $${\varepsilon }_{ik}={\xi }_{i}+{\theta }_{k},$$ where $${\theta }_{k}$$ is the random effect of environment $$k$$ ($$k=1,..{n}_{E})$$ experienced by animal $$i,\left( {{{\varvec{\uptheta}}}\sim MVN\left( {0,{\mathbf{I}}\sigma_{\theta }^{2} } \right)} \right),$$ and $${\xi }_{i}$$ is the random remaining residual of the transmissibility sampling with variance $${\sigma }_{\xi i}^{2}$$. Given this decomposition, the variance of $${\varepsilon }_{ik}$$ (i.e. $${\delta }_{i}{\sigma }_{t}^{2}$$) is decomposed into $$\left({\sigma }_{\xi i}^{2}+{\sigma }_{\theta }^{2}\right),$$ and the covariance between transmissibility samplings of animals $$i$$ and $$i{\prime}$$ sharing the same environment is: $$cov\left({\varepsilon }_{ik},{\varepsilon }_{i{\prime}k}\right)={\sigma }_{\theta }^{2},$$ and 0 elsewhere. $$r=\frac{{\sigma }_{\theta }^{2}}{{\sigma }_{t}^{2}}$$ is defined as the proportion of total transmissibility variance explained by the environmental influence, and $$\rho =\frac{r}{1-{\omega }_{d}^{2}-{\omega }_{s}^{2}}$$ as the correlation between the transmissibility samplings of animals with known parents sharing the same environment (i.e., the maximal correlation between transmissibility samplings that can be obtained). The proportion $$r$$ is positive and upper bounded by $$(1-{\omega }_{d}^{2}-{\omega }_{s}^{2})$$ because $$\rho \le 1$$. The variance of the remaining residual of transmissibility sampling for animal $$i$$ is $${\sigma }_{\xi i}^{2}=\left({\delta }_{i}-r\right){\sigma }_{t}^{2}$$. As a result, the co-variance matrix of $${\varvec{\varepsilon}}$$ is $${\mathbf{D}}_{\mathbf{E}}{\sigma }_{t}^{2},$$ where $${\mathbf{D}}_{\mathbf{E}}$$ can be reorganized as a block diagonal matrix with $${n}_{E}$$ blocks. Each block corresponds to one specific environment. They all have the same matrix structure of various sizes depending on the number of animals sharing the same environment $$k$$: $${\delta }_{i}$$ coefficients on the diagonal and coefficient $$r$$ as off-diagonal entries (see Additional file 1). The transmissibility model including transmitted environmental influences is then the same as Eq. (1), but the transmissibility matrix $$\mathbf{M}$$ is modified. This modified transmissibility matrix is referred to as $${\mathbf{M}}_{{\varvec{E}}}$$. Once again, its inverse can be easily obtained by the decomposition: $${\mathbf{M}}_{\mathbf{E}}^{{\varvec{-1}}}=\mathbf{L}\mathbf{^{\prime}}{\mathbf{D}}_{\mathbf{E}}^{{\varvec{-1}}}\mathbf{L}$$. Shared environmental effects are transmitted from one generation to another through path coefficients of transmission. A detailed description of the way to compute $${\mathbf{M}}_{\mathbf{E}}^{{\varvec{-1}}}$$ is provided in Additional file 1. Thus, in the transmissibility model with environment, compared to the traditional transmissibility model, one additional parameter has to be estimated: $$r$$. These parameters can be estimated with the restricted maximum likelihood method (REML) using ASReml [28] and the OWN Fortran program, freely available on the Zenodo website (10.5281/zenodo.8223572), which we have developed.

Consider the special case where a group of animals experiences a particular shared environment (e.g., a stressful environment induced in an experiment) with effect ${\theta }_{0}{\sigma }_{t}$, while the other animals are each in their own environment, different from each other. In that case, information about the variance of the transmitted environmental effect leads to the difference between the average transmissibility potential of the group of animals experiencing the particular environment and the other animals. Thus, ${\sigma }_{\theta }^{2}={\theta }_{0}^{2}{\sigma }_{t}^{2}$, hence $r={\theta }_{0}^{2}$ and ${\theta }_{0}<\sqrt{(1-{\omega }_{d}^{2}-{\omega }_{s}^{2})}$. The reorganized ${\mathbf{D}}_{\mathbf{E}}$ matrix has only one block, as previously defined, and the remaining matrix is diagonal with ${\delta }_{i}$ terms.

### Simulation study

The aim of the simulation study was to evaluate the performances of the transmissibility model with environment so as to detect and quantify transgenerational transmitted environmental effects and not to confuse them with non-transmissible environmental effects. Indeed, on the one hand, the effect ${\theta }_{k}$ of environment $k$ may be transmissible across generations; in which case it has an effect on the transmissible potential ${t}_{i}$ of animal $i$ experiencing environment $k$ as described above. On the other hand, the effect of the environment may not be transmissible but nonetheless it may have an impact on the phenotype of animal $i$. In this case, the environmental effect can be modeled as a fixed effect of the mixed model used to simulate the phenotype. These two situations were investigated in the simulations.

**Fig. 1:**
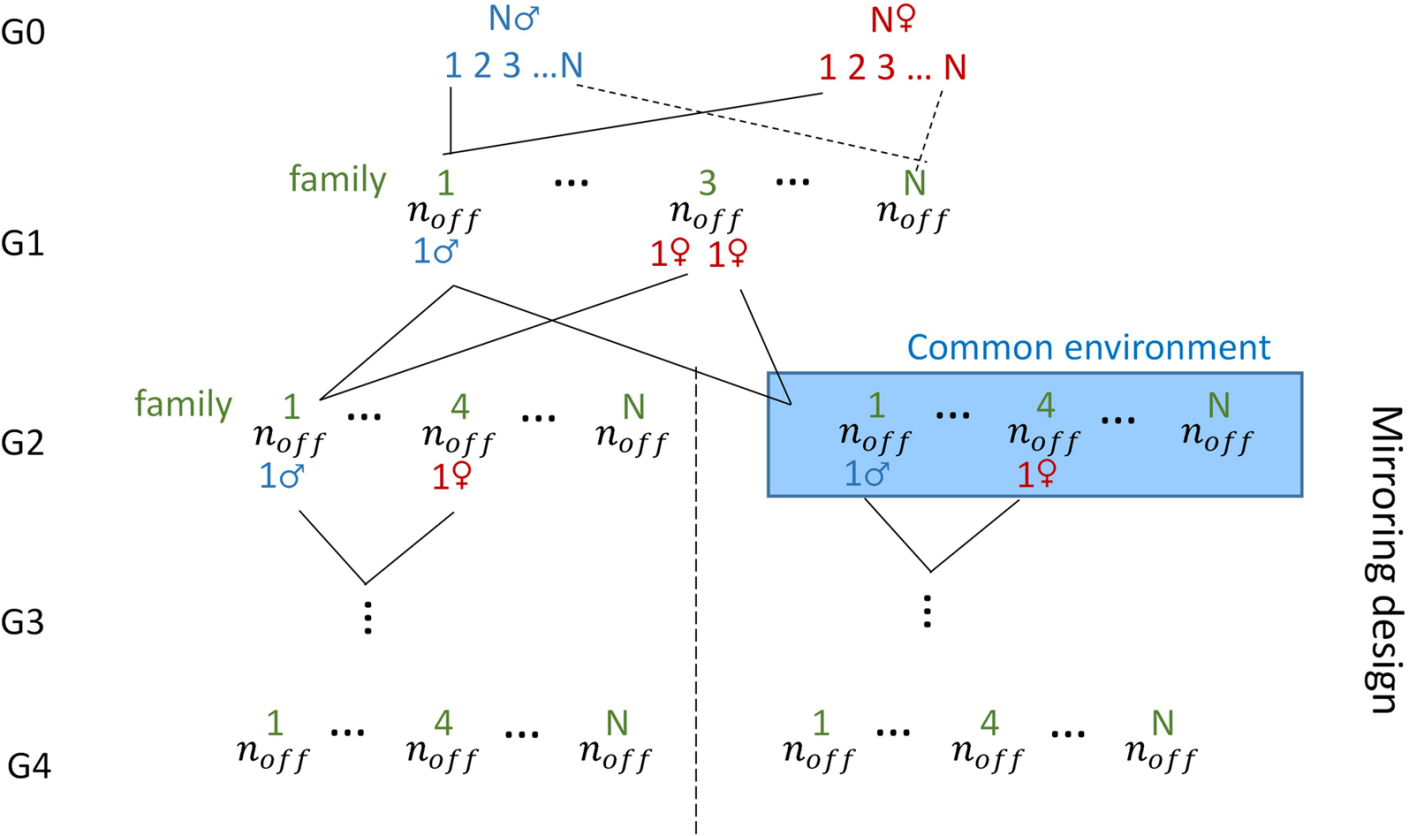
Simulated population in the mirroring design. $N$ is the number of pairs of male and female founders; ${n}_{off}$ is the number of offspring per pair

A population that mimics a mirrored experimental design proposed by Leroux et al. [29] was simulated for the purpose of testing the transmission of environmental effects across generations (Fig. 1). The population consisted of $N$ couples of founders ($N$ families: G0) that gave birth to ${n}_{off}$ offspring each (G1). One male of each family in G1 was then mated to two sisters of another family that then gave birth to two groups of ${n}_{off}$ offspring ($2N{n}_{off}$ animals in G2). Half of the groups (one for each family; $N{n}_{off}$ animals) was then considered as experiencing the same particular environment, for example a stressful environment, (from this point on, the descendants of the two groups are qualified as belonging to two different lines: $E+,E-$). Three generations were then produced for each line with exact parallel pedigrees via mirrored single-pair matings at each generation.

Phenotypes were simulated for all animals, and different scenarios for modeling the impact of the environment were considered. In Scenario 1, the environment has an impact on the phenotype but is not vertically transmitted. It was simulated by adding a constant to the phenotype of animals that experienced the stressful environment; ${y}_{i}={\mathbf{x}}_{\mathbf{i}}{\varvec{\upbeta}}+{\theta }_{i}+{t}_{i}+{e}_{i}$, where ${\theta }_{i}=\sqrt{r}{\sigma }_{t}$ if animal $i$ is in the particular environment and ${\theta }_{i}=$ 0 elsewhere, and ${t}_{i}$ is modeled as in the “classical” transmissibility model. In Scenario 2, the environment has an impact on the transmissible potential (i.e., it is a transmitted environmental effect). This impact was modeled by adding a constant to the transmissible potentials of animals experiencing the stressful environment, i.e., ${t}_{i}={{\omega }_{s}t}_{si}+{{\omega }_{d}t}_{di}+{\theta }_{i}+{\xi }_{i}$, where ${\theta }_{i}=\sqrt{r}{\sigma }_{t}$ if animal $i$ is in the particular environment and ${\theta }_{i}=$ 0 elsewhere, and ${\varvec{\upxi}}$ were independently distributed with variance equal to $\left({\delta }_{i}-r\right){\sigma }_{t}^{2}$ for animals that experienced the particular environment, and ${\delta }_{i}{\sigma }_{t}^{2}$ elsewhere.

The same four sets of parameters were used in the two scenarios (Table 1), the difference between scenarios being how the phenotypes were simulated (transmitted or not transmitted environmental effect). Values of $r$ were chosen in order to correspond to a wide range of values for $\rho$ (from 0.30 to 0.70), the maximal correlation that can be obtained between transmissibility samplings of animals sharing the same environment when the environmental effect is transgenerationally transmitted.

**Table 1:** Description of the sets of parameters used in the simulations

Parameters	Set 1	Set 2	Set 3	Set 4
${\omega }_{s}$	0.40	0.40	0.40	0.20
${\omega }_{d}$	0.25	0.25	0.25	0.50
$r$ $\left(\rho \right)$	0.544 (0.70)	0.389 (0.50)	0.233 (0.30)	0.213 (0.30)

In Scenario 1, the model of simulation is: ${y}_{i}={\mathbf{x}}_{\mathbf{i}}{\varvec{\upbeta}}+{\theta }_{i}+{t}_{i}+{e}_{i},$ where ${\theta }_{i}=\sqrt{r}{\sigma }_{t}$ if animal $i$ is in the particular environment, ${\theta }_{i}=$ 0 elsewhere; and ${t}_{i}$ is modeled as in the “classical” transmissibility model

In Scenario 2: the model of simulation is: ${y}_{i}={\mathbf{x}}_{\mathbf{i}}{\varvec{\upbeta}}+{t}_{i}+{e}_{i},$ where ${t}_{i}={{\omega }_{s}t}_{si}+{{\omega }_{d}t}_{di}+{\theta }_{i}+{\xi }_{i}$, ${\theta }_{i}=\sqrt{r}{\sigma }_{t}$ if animal $i$ is in the particular environment, ${\theta }_{i}=$ 0 elsewhere; $\mathbf{\xi}$ are independently distributed with variance equal to $\left({\delta }_{i}-r\right){\sigma }_{t}^{2}$ for animals that experience the particular environment, and ${\delta }_{i}{\sigma }_{t}^{2}$ elsewhere

${\delta }_{i}=\left(1-{\omega }_{s}^{2}-{\omega }_{d}^{2}\right)$ if both parents are known, $\left(1-{\omega }_{d}^{2}\right)$ for animals of unknown sire; $\left(1-{\omega }_{s}^{2}\right)$ for animals of unknown dam; and 1 for animals for which both parents are unknown. $\rho =\frac{r}{1-{\omega }_{s}^{2}-{\omega }_{d}^{2}}$

The transmissibility model and the transmissibility model with environment were applied to the simulated data of the different scenarios (100 replicates each and for each set of parameters). The environment (cross-classified variable equal to 1 if animals experience the particular environment, 0 elsewhere) was included as a fixed effect in both models. The null hypothesis of “no transmitted environmental effect” (i.e., $r=0$) was tested by comparing the two models using a likelihood ratio test (LRT). Because the test corresponds to a test at the boundary of the parameter space of $r$, the asymptotic distribution of the LRT is a 50:50 mixture, ${\chi }_{0}^{2}$ and ${\chi }_{1}^{2}$ [30]. The null hypothesis was then rejected at the $\alpha$-risk of $5\%$ if the LRT was greater than 2.706. The transgenerational effect of the environment was also assessed, as previously done in the literature, by comparing the phenotypic performance of the two lines in the last generation [29, 31, 32]. The difference in the average phenotype of the two lines in the last generation was tested using a paired T-test at the $\alpha$-risk of 5%. Using these different scenarios and these two models of estimation, our aim was (i) to estimate the realized type I error of the LRT and the paired T-tests when there is an environmental effect but that is not transmissible (Scenario 1); and (ii) to estimate the power of the LRT and the paired T-tests to detect the transmitted environmental effects of different importance and to illustrate the capacity of the transmissibility model with environment to correctly estimate parameters (Scenario 2). For all the simulations, $N=20, {n}_{off}=10,$ leading to 1840 animals in the pedigree. The residual variance was fixed to 10 and the transmissibility variance to 5 in all scenarios.

To illustrate the ability of the transmissibility model with environment to correctly estimate parameters in any population with many different environments, we also performed an additional simulation considering the same population structure as used in David and Ricard [21], 15 different particular environments and the simulation parameters of set 1 (see Additional file 2 for details).

## Results

The number of simulations per set and scenario for which the null hypothesis of no transgenerational environmental effect is rejected using the LRT or the T-test is in Table 2. Evaluated in different situations where there is an environmental effect not transmitted across generations, the realized type I error (average over the different sets) of the LRT was 5%. In Scenario 1, the average difference between phenotypes of the two lines in the last generation was equal to 0.03 ± 0.51. The realized type I error of the paired T-test (average over the different sets) was 14.75%. The power of the transmissibility model with environment to detect transmitted environmental effects was 94, 75, 46 and 45% for sets 1 to 4, respectively. The average difference in phenotype between the two lines in the last generation when the effect of the environment is transmitted across generations was equal to 0.29 ± 0.48, 0.33 ± 0.52, 0.41 ± 0.52 and 0.32 ± 0.55 for sets 1 to 4, respectively. The power to detect differences in average phenotypes between the two lines in the last generation using a paired T-test was equal to 26, 24, 22 and 23% for sets 1 to 4, respectively.

**Table 2:** Number of simulations over 100 rejecting the null hypothesis of no transmitted environmental effect

Set	${\omega }_{s}$	${\omega }_{d}$	$r$ $\left(\rho \right)$	Scenario 1 (without a transmitted environmental effect)		Scenario 2 (with a transmitted environmental effect)	
				LRT	T-test	LRT	T-test
1	0.40	0.25	0.544 (0.70)	7	15	94	26
2	0.40	0.25	0.389 (0.50)	7	15	75	24
3	0.40	0.25	0.233 (0.30)	4	11	46	22
4	0.20	0.50	0.213 (0.30)	2	18	45	23
Average percentage				5.00	14.75	65.00	23.75

In Scenario 1, the model of simulation is: ${y}_{i}={\mathbf{x}}_{\mathbf{i}}{\varvec{\upbeta}}+{\theta }_{i}+{t}_{i}+{e}_{i},$ where ${\theta }_{i}=\sqrt{r}{\sigma }_{t}$ if animal $i$ is in the particular environment, ${\theta }_{i}=$ 0 elsewhere; and ${t}_{i}$ is modeled as in the “classical” transmissibility model

In Scenario 2: the model of simulation is: ${y}_{i}={\mathbf{x}}_{\mathbf{i}}{\varvec{\upbeta}}+{t}_{i}+{e}_{i},$ where ${t}_{i}={{\omega }_{s}t}_{si}+{{\omega }_{d}t}_{di}+{\theta }_{i}+{\xi }_{i}$, ${\theta }_{i}=\sqrt{r}{\sigma }_{t}$ if animal $i$ is in the particular environment, ${\theta }_{i}=$ 0 elsewhere; $\mathbf{\xi}$ are independently distributed with variance equal to $\left({\delta }_{i}-r\right){\sigma }_{t}^{2}$ for animals that experience the particular environment, and ${\delta }_{i}{\sigma }_{t}^{2}$ elsewhere

${\delta }_{i}=\left(1-{\omega }_{s}^{2}-{\omega }_{d}^{2}\right)$ if both parents are known, $\left(1-{\omega }_{d}^{2}\right)$ for animals of unknown sire; $\left(1-{\omega }_{s}^{2}\right)$ for animals of unknown dam; and 1 for animals for which both parents are unknown. $\rho =\frac{r}{1-{\omega }_{s}^{2}-{\omega }_{d}^{2}}$

LRT: likelihood ratio test comparing the transmissibility and the transmissibility model with environment; the null hypothesis is $H0{:}r=0$

T-test: paired T-test comparing the average phenotype of the two lines $(E+,E-)$ in the last generation. The null hypothesis is $H0{:}{\mu }_{E+}={\mu }_{E-}$

Estimations of the parameters obtained with the transmissibility model and the transmissibility model with environment for the different scenarios and sets of parameters are in Table 3. When the effect of the environment is not transmitted across generations (Scenario 1), estimation of the sire and dam path coefficients of transmission were well estimated and did not significantly differ between the transmissibility model and the transmissibility model with environment. Estimations of $r$ ($\rho$) obtained with the transmissibility model with environment in Scenario 1 ranged from 0.07 to 0.08 (from 0.09 to 0.11) depending on the set of parameters, and were never significantly different from 0. When the effect of the environment is transmitted across generations (Scenario 2), transmission path coefficients estimated with the transmissibility model and the transmissibility model with environment were close to their simulated values, regardless of the set of parameters. For all sets of parameters, the transmission path coefficients obtained with the transmissibility model were larger than those estimated with the transmissibility model with environment (p-values of all paired T-tests < 0.01). Estimates of the residual and transmissibility variances obtained with the two models were closer to their true values in Scenario 2 compared to Scenario 1. The residual variance estimates did not significantly differ between the two models, while the transmissibility variance estimates were larger in the transmissibility model with environment for all sets of parameters (p-values of all paired T-tests < 0.001). This difference increased with the magnitude of the transmitted effect. For instance, the difference between the transmissibility variance estimates was 0.38 in Set 1 ($\rho =0.7$) and 0.18 in Set 3 ($\rho =0.3$). Estimations of $r$ ($\rho$) obtained with the transmissibility model with environment were close to their true values, regardless of the set of parameters. Nonetheless, it should be noted that the standard deviations associated with these estimates were large. Estimations of $r$ obtained in the additional simulation design that considered a real-world population and 15 particular environments were also close to its true value (0.53, simulated value 0.544) and associated with large standard deviation (0.14, for all the results, see Additional file 2).

**Table 3:** Estimates (± sd) obtained with the transmissibility model and the transmissibility model with environment

Set	Simulated value		Scenario 1 (without a transmitted environmental effect)		Scenario 2 (with a transmitted environmental effect	
			Trans	TransEnv	Trans	TransEnv
1	${\sigma }_{e}^{2}$	10	9.64 ± 1.71	9.62 ± 1.73	9.82 ± 1.43	9.68 ± 1.37
	${\sigma }_{t}^{2}$	5	5.33 ± 1.72	5.40 ± 1.75	4.91 ± 1.43	5.29 ± 1.46
	${\omega }_{s}$	0.40	0.39 ± 0.08	0.39 ± 0.08	0.40 ± 0.07	0.38 ± 0.07
	${\omega }_{d}$	0.25	0.25 ± 0.08	0.24 ± 0.08	0.27 ± 0.09	0.24 ± 0.07
	$r$ $(\rho )$	0.544 (0.70)	–	0.08 ± 0.15 (0.10 ± 0.20)	–	0.53 ± 0.18 (0.68 ± 0.25)
2	${\sigma }_{e}^{2}$	10	9.64 ± 1.71	9.62 ± 1.73	9.79 ± 1.47	9.69 ± 1.50
	${\sigma }_{t}^{2}$	5	5.33 ± 1.72	5.40 ± 1.75	5.01 ± 1.49	5.29 ± 1.58
	${\omega }_{s}$	0.40	0.39 ± 0.08	0.39 ± 0.08	0.40 ± 0.07	0.39 ± 0.07
	${\omega }_{d}$	0.25	0.25 ± 0.08	0.24 ± 0.08	0.26 ± 0.09	0.25 ± 0.08
	$r$ $(\rho )$	0.389 (0.50)	–	0.08 ± 0.15 (0.10 ± 0.20)	–	0.39 ± 0.20 (0.51 ± 0.27)
3	${\sigma }_{e}^{2}$	10	9.76 ± 1.73	9.73 ± 1.75	9.83 ± 1.54	9.76 ± 1.55
	${\sigma }_{t}^{2}$	5	5.16 ± 1.79	5.23 ± 1.80	5.00 ± 1.59	5.18 ± 1.65
	${\omega }_{s}$	0.40	0.39 ± 0.08	0.39 ± 0.08	0.40 ± 0.08	0.39 ± 0.08
	${\omega }_{d}$	0.25	0.25 ± 0.08	0.24 ± 0.07	0.25 ± 0.08	0.24 ± 0.08
	$r$ $(\rho )$	0.233 (030)		0.07 ± 0.15 (0.09 ± 0.20)		0.24 ± 0.19 (0.32 ± 0.26)
4	${\sigma }_{e}^{2}$	10	9.86 ± 1.37	9.88 ± 1.36	9.95 ± 1.26	9.90 ± 1.27
	${\sigma }_{t}^{2}$	5	5.07 ± 1.39	5.09 ± 1.39	4.89 ± 1.29	5.05 ± 1.32
	${\omega }_{s}$	0.20	0.20 ± 0.08	0.20 ± 0.08	0.20 ± 0.08	0.20 ± 0.08
	${\omega }_{d}$	0.50	0.49 ± 0.09	0.49 ± 0.09	0.50 ± 0.09	0.49 ± 0.09
	$r$ $(\rho )$	0.213 (0.30)	–	0.07 ± 0.13 (0.11 ± 0.20)	–	0.23 ± 0.17 (0.34 ± 0.26)

In Scenario 1, the model of simulation is: ${y}_{i}={\mathbf{x}}_{\mathbf{i}}{\varvec{\upbeta}}+{\theta }_{i}+{t}_{i}+{e}_{i},$ where ${\theta }_{i}=\sqrt{r}{\sigma }_{t}$ if animal $i$ is in the particular environment, ${\theta }_{i}=$ 0 elsewhere; and ${t}_{i}$ is modeled as in the “classical” transmissibility model

In Scenario 2: the model of simulation is: ${y}_{i}={\mathbf{x}}_{\mathbf{i}}{\varvec{\upbeta}}+{t}_{i}+{e}_{i},$ where ${t}_{i}={{\omega }_{s}t}_{si}+{{\omega }_{d}t}_{di}+{\theta }_{i}+{\xi }_{i}$, ${\theta }_{i}=\sqrt{r}{\sigma }_{t}$ if animal $i$ is in the particular environment, ${\theta }_{i}=$ 0 elsewhere; $\mathbf{\xi}$ are independently distributed with variance equal to $\left({\delta }_{i}-r\right){\sigma }_{t}^{2}$ for animals that experience the particular environment, and ${\delta }_{i}{\sigma }_{t}^{2}$ elsewhere

${\delta }_{i}=\left(1-{\omega }_{s}^{2}-{\omega }_{d}^{2}\right)$ if both parents are known, $\left(1-{\omega }_{d}^{2}\right)$ for animals of unknown sire; $\left(1-{\omega }_{s}^{2}\right)$ for animals of unknown dam; and 1 for animals for which both parents are unknown. $\rho =\frac{r}{1-{\omega }_{s}^{2}-{\omega }_{d}^{2}}$

*Trans* transmissibility model, *TransEnv* transmissibility model with environment

## Discussion

Initial investigations to demonstrate the existence of a transgenerational environmental effect based on phenotypic observations often consist in comparing the phenotypic means of two groups of animals whose ancestors have experienced different environmental conditions [29, 31, 32]. The main drawback of such comparisons is that the phenotypic difference observed between the two groups may be the result of genetic (and/or transmissibility) drift, even if special attention has been devoted to limiting these differences by using a mirrored design, as in Leroux et al. [29]. This phenomenon is illustrated in the current study by the realized type I error of the paired T-test that was higher than the expected 5%. Contrary to what might be expected, comparing phenotypes corrected for additive genetic effects using pedigree information is not a feasible alternative because, if there is a transmissible environmental effect, the additive genetic values will include this effect in their (best linear unbiased) predictions, which will then no longer be detectable by comparing corrected phenotypes. To illustrate this hypothesis, we reran the scenarios for Set 1 (100 simulations) and compared the phenotypes of the two groups using a paired T-test, corrected for transmissibility potential obtained with the transmissibility model (same predictions as an animal model; see David and Ricard [21]). The realized type I error was 2%, but the power to detect transmitted environmental effects declined to 3% due to the absence of any remaining difference between lines in the last generation, which was absorbed in the transmissible potential prediction.

The results of the simulations showed that the power of the T-test to detect transmitted environmental effects was small (< 26%). Since the non-genetic transmissible factors, which are at the origin of the transgenerational impact of the environment [33–35], are diluted in future generations [23], the difference between lines decreases by a multiplication factor equal to ${\omega }_{s}+{\omega }_{d} \left(<1\right)$ at each generation. In the present study, the difference between lines in the last generation corresponded to 27 and 34% of the initial difference for Sets 1 to 3 and Set 4, respectively, and was thus difficult to highlight using the T-test. To estimate transmitted environmental effects, the transmissibility model with environment uses covariance information within lines of all generations, which explains the higher power of the LRT to detect transmitted environmental effects compared to the T-test. Since the LRT consists in testing if $r=0$, the power to detect transmitted environmental effects increased (quasi-linearly) with the value of $r$. Standard errors associated with $\widehat{r}$ were large, indicating that a sufficiently large number of phenotyped individuals is needed to be able to detect transmitted environmental effects. Given the dilution effect, it is preferable to favor a large number of animals per generation rather than a large number of generations following the environmental exposure. However, this recommendation is relevant when the origin of the multigenerational environmental impact is the vertical transmission of non-genetic factors only, as simulated in the present study. This corresponds to the mechanisms of the multigenerational impact of the environment mediated by the culture or the microbiota. When the epigenetic mechanisms are at the origin of the transmission of the impact of the environment across generations, the situation becomes a little more complicated. In that case, the multigenerational effect of the environment may be an intergenerational and/or transgenerational effect [36]. In other words, the environment has a direct impact on the animal that experiences the environment, as well as on the subsequent generations whose genetic material was present at the time of exposure due to the direct effects of the parent’s environment/physiology on the developing embryo/fetus or on germ cells. The “direct” environmental impact may thus be different depending on the generation. To account for such situations in the transmissibility model with environment, two (or three) different effects of the environment must be considered depending on the generation (direct exposure or exposure at the level of the embryo or the germ cells), leading to covariance between transmissibility samplings of same-stage animals (born, embryo, germ cells) that experience the environment. This can be implemented in the transmissibility model with environment by considering two (or three) different environments, one for each stage. Further investigations are needed to evaluate the quality of the estimations in such situations. It should be noted that the covariance between the transmissibility samplings of animals sharing the same environment does not only account for the impact of the transmissible environmental effect common to these animals but also for the horizontal transmission of non-genetic effects between these animals reported for culture and microbiota [24, 25]. When applying the transmissibility model with environment, it is possible to include a random genetic effect in addition to the transmissibility potential in order to distinguish genetic from non-genetic factors. However, this will certainly lead to practical identifiability issues as described for the transmissibility model [21] and was therefore not investigated in this study. To solve this practical identifiability problem, it is necessary to have additional information on non-genetic inherited factors such as direct measurements of microbiota, methylation and to apply the transmissibility model with environment using these direct measurements as proposed by David et al. [37]. It will then be possible to dissociate the different heritable factors in a mixed model in a second step, considering the path coefficients of transmission and $r$ known for each of them, provided they are sufficiently different between the factors. This approach using complementary information also has the advantage of identifying which non-genetic factor is the transgenerational transmission vector of environmental effects.

To estimate the parameters of the transmissibility model with environment, it is necessary to compute the inverse of the block diagonal matrix ${\mathbf{D}}_{\mathbf{E}}$. In the program that we propose, this inverse is computed, by blocks that use the formula proposed by Searle [38] (see Additional file 1) that is applicable on matrices for which all diagonal elements are equal. Thus, it is necessary that parental information be the same for all of the animals that experience the particular environment (all have both parents known, one same parent known or unknown parents). This limitation is inherent to the method used to obtain the inverse of the covariance matrix in the estimation program, but not to the transmissibility model itself. A generalization is certainly possible but would probably require very large computation times.

The results obtained on simulated data illustrated the good capacities of the transmissibility model with environment to correctly estimate variances, covariances and path coefficients of transmission in the presence and absence of transmitted environmental effects. The simulated design corresponded to an experimental design dedicated to the test of transmitted environmental effects based on a T-test. The transmissibility model with environment can nevertheless be applied in a population without this particular structure and, if the program we propose is used, as long as the animals in the same environment have the same type of parental information as described in Additional file 2. The proportion of transmitted variance used for the simulation was moderate (30%) and the sire and dam transmissibilities were small to moderate depending on the set (0.08 and 0.16 for the dam transmissibility, 0.064 and 0.13 for the sire transmissibility). The different values of the transmitted environmental effect used in the simulations corresponded to moderate to large correlations between transmissibility samplings of the animals sharing the same environment. Our knowledge of the true magnitude of these transmissible environmental effects is limited, but since the predicted transmissible potential from the observed phenotypes is a weighted sum of the different sources of inheritance, the impact of the transmissible environment may be even smaller than this due to the relative proportion of environmentally insensitive genetics in the transmissibility. To ensure sufficient power of detection of the transmissible environment from the observed phenotypes, a larger population size is likely to be required. If the information is available, another solution is to test the impact of transmitted environmental effects with the transmissibility model with environment using the direct measurements of the non-genetic inherited factors, as previously described. In any case, the transmissibility model with environment, as currently programmed, is not intended to be applied routinely to large populations, due to the long computation times it requires. The CPU time for one iteration of convergence on a linux system and intel®Xeon®E5-2698v3 processor for 2525 individuals in the pedigree and 15 environments averaged 900 s. The model, as currently programmed, is not intended to be used routinely for large datasets, but rather to detect the existence of transmissible environmental effects, so that further investigations can be carried out to understand (and perhaps control) this transmission.

## Conclusions

The transmissibility model with environment is an effective model to detect transmitted environmental effects. Thus, it offers a new tool to assess the importance of non-genetic factors in the form of traits. This could lead to a rethinking of classical genetic selection into adaptive selection by acting on the environment of future reproducers.

### Supplementary Information

**Additional file 1. **Inverse of the transmissibility matrix with transmitted environmental effect. Description of the method used to compute the inverse of the transmissibility matrix.

**Additional file 2. **Simulation in a non-structured population with 15 different environments. Description of an additional simulation study consisting in a non-structured population with 15 different environments.

## Data Availability

The authors state that all information necessary to perform the simulation study presented in the manuscript is fully represented within the manuscript. The code source and tutorial to apply the transmissibility model with environment are available on the Zenodo website, 10.5281/zenodo.8223572.
