# Supplementary material for: An improved transmissibility model to detect transgenerational transmitted environmental effects
Source: Genet Sel Evol. 2023 Sep 21;55:66. doi: 10.1186/s12711-023-00833-y (PMC10512618; doi:10.1186/s12711-023-00833-y)
Supplement: Supplementary file 1 — Additional file 1. Inverse of the transmissibility matrix with transmitted environmental effect. Description of the method used to compute the inverse of the transmissibility matrix. [file 12711_2023_833_MOESM1_ESM.pdf]

Additional file 1: inverse of the transmissibility matrix with transmitted environmental effect

For animal i, born from sire si and dam di, $t_i = \omega_s t_{si} + \omega_d t_{di} + \varepsilon_i$.

In matrix notation $\mathbf{t} = \boldsymbol{\omega}\mathbf{t} + \boldsymbol{\varepsilon}$, where, if animals are sorted from oldest to youngest in the pedigree, $\boldsymbol{\omega}$ is a lower triangular matrix with path coefficients of transmission as off diagonal entries, $\mathbf{V}(\boldsymbol{\varepsilon}) = \mathbf{D_E}\sigma_t^2$ where $\mathbf{D_E}$ is a square matrix with δ_i coefficients on the diagonal and coefficient r as off-diagonal entries between animals sharing the same environment, 0 elsewhere. Thus $\mathbf{t} = [\mathbf{I} - \boldsymbol{\omega}]^{-\mathbf{1}}\boldsymbol{\varepsilon}$, therefore $\mathbf{M_E} = \mathbf{V}(\mathbf{t}) = (\mathbf{I} - \boldsymbol{\omega})^{-\mathbf{1}}\mathbf{V}(\boldsymbol{\varepsilon})(\mathbf{I} - \boldsymbol{\omega}')^{-\mathbf{1}}$ and then $\mathbf{M}_{\boldsymbol{E}}{}^{-1} = (\mathbf{I} - \boldsymbol{\omega}')\mathbf{V}(\boldsymbol{\varepsilon})^{-\mathbf{1}}(\mathbf{I} - \boldsymbol{\omega}) = \mathbf{L}'\mathbf{D}_{\boldsymbol{E}}{}^{-\mathbf{1}}\mathbf{L}$**,** where **L** is a lower triangular matrix with 1's on the diagonal and the negatives of the sire and dam coefficients of transmission as off-diagonal entries. Thus the difficulty is to compute $\mathbf{D}_{\boldsymbol{E}}{}^{-\mathbf{1}}$**.** For simplicity, we consider the simple case of one particular environment shared by n individuals in a population of N individuals. By reordering the animals into 2 groups, first group: the $N - n$ animals not sharing the same environment and second group the n animals sharing the same environment, the reordered $\mathbf{D}_{\boldsymbol{E}}$ matrix is a block diagonal matrix with two blocks, $\mathbf{B_1}$ and $\mathbf{B_2}$. Blocks $\mathbf{B_1}$ is a diagonal matrix with variances of ε_t relative to σ_t^2 (δ_i) as components, and block $\mathbf{B_2}$, corresponding to the n animals sharing the same environment is a dense matrix with variances of ε_t relative to σ_t^2 on the diagonal and the covariance between ε_t relative to σ_t^2 as off-diagonal entries (r). Under the assumption that all animals in $\mathbf{B_2}$ have similar known parental information (i.e., all have both parents unknown or all sires known or all dams known or all parents known), it can be rewritten as:

$\mathbf{B_2} = \begin{bmatrix} \delta & r & \cdots & r \\ r & \delta & \cdots & r \\ \vdots & \vdots & \ddots & \vdots \\ r & r & \cdots & \delta \end{bmatrix}$ where $\delta = (1 - \omega_s^2 - \omega_d^2)$ if both parents are known, $(1 - \omega_d^2)$ for animals of unknown sire, $(1 - \omega_s^2)$ for animals of unknown dam, and 1 for animals for which both parents are unknown. The inverse of the ordered $\mathbf{D}_{\boldsymbol{E}}$ is then easily obtained by a by-block

inversion with, from Searle [38]: $\mathbf{B_2^{-1}} = \frac{1}{\delta - r}\mathbf{I_n} + \frac{-r}{(\delta - r)(\delta + (n-1)r)}\mathbf{J_n}$. The extension to multiple environments is straightforward.

As an example, let's consider the following pedigree where animals 5, 6 and 7 are experiencing the same particular environment and consider that $\omega_s = 0.3, \omega_d = 0.5, r = 0.2$

Animal	Sire	Dam	Sex	Environement
1	0	0	Sire	0
2	0	0	Dam	0
3	0	0	Dam	0
4	1	2	Sire	0
5	1	2	Dam	1
6	1	3	Sire	1
7	1	3	Dam	1
8	0	5	Dam	0
9	6	5	Dam	0
10	4	7	Dam	0

$$\mathbf{L} = \begin{bmatrix} 1 & & & & & & & & & \\ 0 & 1 & & & & & & & & \\ 0 & 0 & 1 & & & & & & 0 & \\ -0.3 & -0.5 & 0 & 1 & & & & & & \\ -0.3 & -0.5 & 0 & 0 & 1 & & & & & \\ -0.3 & 0 & -0.5 & 0 & 0 & 1 & & & & \\ -0.3 & 0 & -0.5 & 0 & 0 & 0 & 1 & & & \\ 0 & 0 & 0 & 0 & -0.5 & 0 & 0 & 1 & & \\ 0 & 0 & 0 & 0 & -0.5 & -0.3 & 0 & 0 & 1 & \\ 0 & 0 & 0 & -0.3 & 0 & 0 & -0.5 & 0 & 0 & 1 \end{bmatrix}$$

The **D** matrix not considering shared environment (classical transmissibility model) is

$$\mathbf{D} = \begin{bmatrix} 1 & & & & & & & & & \\ 0 & 1 & & & & & & & & \\ 0 & 0 & 1 & & & & & Symm & & \\ 0 & 0 & 0 & 0.66 & & & & & & \\ 0 & 0 & 0 & 0 & 0.66 & & & & & \\ 0 & 0 & 0 & 0 & 0 & 0.66 & & & & \\ 0 & 0 & 0 & 0 & 0 & 0 & 0.66 & & & \\ 0 & 0 & 0 & 0 & 0 & 0 & 0 & 0.75 & & \\ 0 & 0 & 0 & 0 & 0 & 0 & 0 & 0 & 0.66 & \\ 0 & 0 & 0 & 0 & 0 & 0 & 0 & 0 & 0 & 0.66 \end{bmatrix}$$

while the $\mathbf{D_E}$ matrix accounting for transmitted environmental effect would be

$$\mathbf{D_E} = \begin{bmatrix} 1 & & & & & & & & & \\ 0 & 1 & & & & & & & & \\ 0 & 0 & 1 & & & & & & Symm & \\ 0 & 0 & 0 & 0.66 & & & & & & \\ 0 & 0 & 0 & 0 & 0.66 & & & & & \\ 0 & 0 & 0 & 0 & 0.2 & 0.66 & & & & \\ 0 & 0 & 0 & 0 & 0.2 & 0.2 & 0.66 & & & \\ 0 & 0 & 0 & 0 & 0 & 0 & 0 & 0.75 & & \\ 0 & 0 & 0 & 0 & 0 & 0 & 0 & 0 & 0.66 & \\ 0 & 0 & 0 & 0 & 0 & 0 & 0 & 0 & 0 & 0.66 \end{bmatrix}$$

Given Searles, $\mathbf{D_E^{-1}}[\mathbf{5{:}7,5{:}7}] = \frac{1}{0.46}\mathbf{I_n} + \frac{-0.2}{(0.46)(1.06)}\mathbf{J_n}$

Considering that $\mathbf{M}_E = \left(\mathbf{L}'\mathbf{D}_E{}^{-\mathbf{1}}\mathbf{L}\right)^{-\mathbf{1}}$ and $\mathbf{M} = (\mathbf{L}'\mathbf{D}^{-\mathbf{1}}\mathbf{L})^{-\mathbf{1}}$, matrices $\mathbf{M}$ and $\mathbf{M}_E$ are :

$$\mathbf{M} = \begin{bmatrix} 1 & & & & & & & & & \\ 0 & 1 & & & & & & & & \\ 0 & 0 & 1 & & & & & & Symm & \\ 0.3 & 0.5 & 0 & 1 & & & & & & \\ 0.3 & 0.5 & 0 & 0.34 & 1 & & & & & \\ 0.3 & 0 & 0.5 & 0.09 & 0.09 & 1 & & & & \\ 0.3 & 0 & 0.5 & 0.09 & 0.09 & 0.34 & 1 & & & \\ 0.15 & 0.25 & 0 & 0.17 & 0.50 & 0.05 & 0.05 & 1 & & \\ 0.24 & 0.25 & 0.15 & 0.20 & 0.53 & 0.35 & 0.15 & 0.26 & 1.03 & \\ 0.24 & 0.15 & 0.25 & 0.35 & 0.15 & 0.20 & 0.53 & 0.07 & 0.13 & 1.03 \end{bmatrix}$$

$$\mathbf{M_E} = \begin{bmatrix} 1 & & & & & & & & & \\ 0 & 1 & & & & & & & & \\ 0 & 0 & 1 & & & & & & Symm & \\ 0.3 & 0.5 & 0 & 1 & & & & & & \\ 0.3 & 0.5 & 0 & 0.34 & 1 & & & & & \\ 0.3 & 0 & 0.5 & 0.09 & \mathbf{0.29} & 1 & & & & \\ 0.3 & 0 & 0.5 & 0.09 & \mathbf{0.29} & \mathbf{0.54} & 1 & & & \\ 0.15 & 0.25 & 0 & 0.17 & 0.50 & \mathbf{0.15} & \mathbf{0.15} & 1 & & \\ 0.24 & 0.25 & 0.15 & 0.20 & \mathbf{0.59} & \mathbf{0.45} & \mathbf{0.31} & \mathbf{0.29} & 1.09 & \\ 0.24 & 0.15 & 0.25 & 0.35 & \mathbf{0.25} & \mathbf{0.30} & 0.53 & \mathbf{0.12} & \mathbf{0.21} & 1.03 \end{bmatrix}$$
