# Supplementary material for: An improved transmissibility model to detect transgenerational transmitted environmental effects
Source: Genet Sel Evol. 2023 Sep 21;55:66. doi: 10.1186/s12711-023-00833-y (PMC10512618; doi:10.1186/s12711-023-00833-y)
Supplement: Supplementary file 2 — Additional file 2. Simulation in a non-structured population with 15 different environments. Description of an additional simulation study consisting in a non-structured population with 15 different environments. [file 12711_2023_833_MOESM2_ESM.pdf]

Additional file 2: Simulation in a non-structured population with 15 different environments

Datasets consisted of a four-generation population. The first generation comprised 25 sires and 100 dams that were randomly mated to give birth to 800 offspring (8 offspring per dam, sex ratio = 1/2). Among the progeny, 25 males and 100 females were sampled randomly to be the parents of the next generation. Same process was repeated for each generation. The final population comprised 2525 individuals. 90% of the animals of the second generation were randomly assigned to 15 different environments. The random effect θ_k of environment k (k=1,..15) was sampled in ($\boldsymbol{\theta} \sim N\left(0, \mathbf{I}\sigma_\theta^2\right)$). Phenotypes were simulated for all animals in the pedigree according to scenario 2, i.e. for animal i experiencing particular environment k; $y_{ik} = \mathbf{x_i}\boldsymbol{\beta} + t_{ik} + e_i$, with $t_{ik} = \omega_s t_{si} + \omega_d t_{di} + \theta_k + \xi_i$ and $\boldsymbol{\xi}$ were independently distributed with variance equal to $(\delta_i - r)\sigma_t^2$ for animals that experienced one of the 15 particular environments, and $\delta_i \sigma_t^2$ elsewhere. Same parameters as those of set1 where used for the simulation. Parameters were estimated by applying the transmissibility model with environment to the simulated phenotypes in 125 replicates. Mean and S.D of estimates are provided in the table below.

parameters	ω_s	ω_d	r	σ_t^2	σ_e^2
Simulated	0.40	0.25	0.544	5	10
estimated	0.39 ± 0.07	0.25 ± 0.06	0.53 ± 0.14	5.1 ± 1.3	9.9 ± 1.2
